# B-Cell-Depleting Therapy Improves Myocarditis in Seronegative Eosinophilic Granulomatosis with Polyangiitis

**DOI:** 10.3390/jcm10194577

**Published:** 2021-10-02

**Authors:** Chrong-Reen Wang, Yi-Shan Tsai, Hung-Wen Tsai, Cheng-Han Lee

**Affiliations:** 1 Division of Rheumatology, Department of Internal Medicine, National Cheng Kung University Hospital, Tainan 70403, Taiwan; 2 Department of Medical Imaging, National Cheng Kung University Hospital, Tainan 70403, Taiwan; n506356@gmail.com; 3 Department of Pathology, National Cheng Kung University Hospital, Tainan 70403, Taiwan; hungwen@mail.ncku.edu.tw; 4 Division of Cardiology, Department of Internal Medicine, National Cheng Kung University Hospital, Tainan 70403, Taiwan; appollolee@hotmail.com

**Keywords:** eosinophilic granulomatosis with polyangiitis, overt myocarditis, seronegative, B-cell-depleting therapy, IL-5-mediated eosinophilia

## Abstract

Cardiac involvement is a major mortality cause in eosinophilic granulomatosis with polyangiitis (EGPA), requiring novel therapeutics to spare the use of cyclophosphamide with known cardiotoxicity. Despite the observed efficacy of B-cell-depleting therapy in myocarditis of seropositive microscopic polyangiitis, it remains to be elucidated in seronegative EGPA. A retrospective study was performed in 21 hospitalized active patients aged 20 to 70 years with five-factor score 1 or 2, eosinophil counts 10,034 ± 6641/μL and vasculitis scores 27 ± 6. Overt myocarditis was identified in 10 cases, at disease onset in 6 and relapse in 4, with endomyocarditis in 4 and myopericarditis in 4. Five seronegative and one seropositive patient received rituximab with an induction regimen 375 mg/m^2^ weekly × 4 for refractory or relapse disease, and the same regimen for annual maintenance therapy. All cases had lower eosinophil counts, improved cardiac dysfunction and clinical remission with a relapse-free follow-up, 48 ± 15 months after the induction treatment. One seronegative endomyocarditis patient had eosinophilia and disease relapse with asthma attack and worsening cardiac insufficiency 24 months after induction, achieving clinical remission under anti-IL-5 therapy. Our findings suggest the suppression of IL-5-mediated eosinophilia as an action mechanism of B-cell-depleting therapy in seronegative EGPA myocarditis.

## 1. Introduction

Eosinophilic granulomatosis with polyangiitis (EGPA), a rare autoimmune disorder occurring exclusively in asthmatic patients, is classified as anti-neutrophil cytoplasmic antibody (ANCA)-associated vasculitis (AAV) [1,2]. Among the three AAV-related disorders, cardiac involvement presenting as myocarditis is most commonly observed in EGPA with serious complications, including overt heart failure and sudden cardiac death [3,4]. AAV therapy can be adapted for the presence of poor prognostic factors, i.e., five-factor score (FFS) [1,5]. In major organ damage with the presence of FFS-like cardiac insufficiency, in addition to initial high-dose corticosteroids (CS), cyclophosphamide (CYC) is usually prescribed to induce disease remission [1,6]. Nevertheless, the majority of deaths in EGPA are attributed to disease activity with heart involvement in spite of combined CS and CYC therapy [3,6,7]. Furthermore, given the significant toxicity of CYC, it is generally acknowledged not to exceed 10 g to 15 g of exposure, due to the dosages-related cardiotoxicity [7], let alone the increased risks of infertility, infections and malignancies. Apparently, there is a need for novel therapeutics to efficiently control the disease activity and spare the use of CYC [8].

Eosinophils play a central role in the EGPA pathophysiology with blood and tissue eosinophilia as the disease hallmark; IL-5 is recognized as the key mediator in the development and maintenance of this pathogenic finding [8,9]. Through the presentation of autoantigens and costimulatory signaling to Th2 cells, B cells can participate in the pathogenesis by inducing the release of IL-5, resulting in the activation, maturation, survival and recruitment of eosinophils [6,7,8,9,10]. Therapeutic indications of rituximab (RTX), a B-cell-depleting anti-CD20 monoclonal antibody (mAb), have been approved for induction therapy in AAV-related diseases, not including EGPA [1,8]. This biologic agent is recommended at induction for active or relapsing disease in severe EGPA [11,12]. Nevertheless, its action mechanisms remain to be elucidated in ANCA-negative patients [13]. Current evidence supports the use of an anti-IL-5 mAb, mepolizumab (MEP), for the induction treatment of active or relapsing EGPA with eosinophilia [8,11,14]. Moreover, the therapeutic efficacy of RTX in overt myocarditis was observed in microscopic polyangiitis, another seropositive AAV disorder [15,16]. In hypereosinophilic syndrome (HES), MEP is recommended to be used in the cardiac manifestation, eosinophilic myocarditis [17]. These findings implicate beneficent effects of B-cell-depleting therapy on ANCA-positive AAV and -negative EGPA myocarditis by potential action mechanisms through reducing autoantibody and IL-5 production, respectively.

This investigation focused on overt myocarditis, an important cause of EGPA mortality, and the therapeutic efficacy of RTX in this manifestation. 

## 2. Materials and Methods

### 2.1. Patients Enrollment

Hospitalized patients, at least 16 years of age to exclude childhood-onset EGPA and fulfilling the 1990 American College of Rheumatology (ACR) criteria for the EGPA classification [18], were analyzed from July 2005 to June 2020. Overt myocarditis was diagnosed, according to the presence of the following criteria: (1) presenting symptoms consistent with heart failure, (2) raised concentrations of cardiac biomarkers, and (3) new or worsening changes on transthoracic echocardiography (TTE) or cardiac magnetic resonance imaging (cMRI), including impaired left ventricle ejection fraction (LVEF, mild 46 to 55%, moderate 30 to 45%, severe below 30%) or plus wall motion abnormality [16,19]. The concurrent pericarditis in addition to myocarditis, i.e., myopericarditis, and the coexistent endocarditis identified by post-gadolinium T1-weighted images in addition to myocarditis, i.e., endomyocarditis, were detected by cMRI or plus TTE. Cardiac rhythm was surveyed by 12-lead electrocardiography and 24-h Holter monitor. Exclusion criteria were coronary artery disease with characterized image findings in coronary angiography or cMRI, viral myocarditis with a confirmed infection history, or preexisting heart diseases under medical therapy [16,19]. The Institutional Review Board approved this study and waived the requirement of informed consents from patients.

### 2.2. Data Collection

Demographical, clinical, laboratory, imaging and pathological data were analyzed, including age/sex, EGPA clinical manifestations, the Birmingham Vasculitis Activity Score (BVAS) [20], FFS [5], myocarditis-related symptoms, the New York Heart Association Functional Classification (NYHAFC), ANCA detected by enzyme-linked immunosorbent assay (ELISA)/immunofluorescence (IF), peripheral blood eosinophil counts, C-reactive protein (CRP) values, IgE levels, circulating B-cell numbers (CD19-positive lymphocytes analyzed by flow cytometry), concentrations of cardiac biomarkers with cardiac troponin I (cTnI), creatine kinase-MB (CKMB) and N-terminal pro-brain natriuretic peptide (NT-proBNP), results of TTE/cMRI and cardiac rhythm, and pathological findings. Medications for disease activity included CS, immunosuppressants (IS) with azathioprine (AZ), CYC and methotrexate (MTX), and RTX. Therapeutics for cardiac involvement included cardiac supportive agents (CSA) for heart failure with angiotensin converting enzyme inhibitor (ACEI), angiotensin-receptor blocker (ARB), beta blocker, diuretic and inotrope, and antiarrhythmics (ART) for cardiac dysrhythmia with sodium or potassium channel blocker and beta blocker. A complete remission was the absence of disease activity corresponding to zero BVAS, while a partial remission was a reduction of no less than 50% in BVAS as compared with the baseline scores [21]. 

### 2.3. Statistical Analyses

Data were expressed as the mean and standard deviation. Numerical data between two groups were compared by the Mann–Whitney test. BVAS, CRP levels and eosinophil counts before and after RTX therapy were calculated by the Wilcoxon signed rank test. A *p* value of less than 0.05 was considered significant in this study.

## 3. Results

### 3.1. EGPA Characteristics

**Figure 1:**
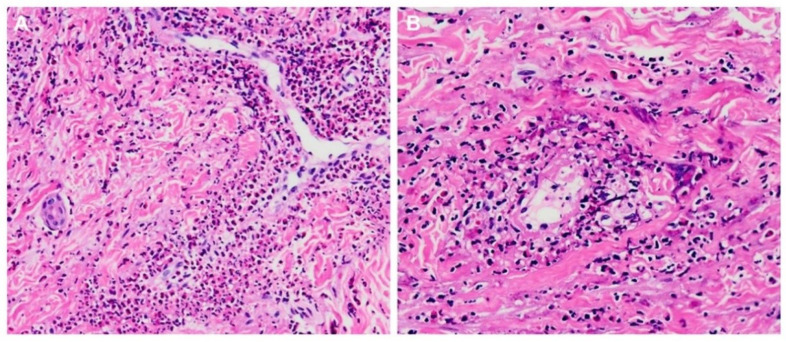
Histopathological findings of tissue eosinophilia and vasculitis changes in skin biopsy specimens from case no. 1. (**A**) Interstitial infiltration of abundant eosinophils in the dermis. Hematoxylin and eosin stain 100×. (**B**) Necrotizing vasculitis in a small vessel with marked infiltration of eosinophils in the vessel wall. Hematoxylin and eosin stain 200×.

In Table 1, the demographic, clinical, laboratory, pathological, medication and outcome profiles of 21 admitted EGPA patients are shown, all with the Han Chinese ethnicity. Histopathological findings of tissue eosinophilia were found in all, or plus vasculitis of small- to medium-sized vessels in 9 patients (Figure 1). The presence of asthma is a characteristic diagnostic feature of EGPA [22], and all enrolled patients had recurrent asthmatic attacks. Other organ involvement and medication profiles during the disease course are outlined in Table 1. At EGPA onset, six patients (case nos. 3, 4, 5, 8, 9 and 10) with heart involvement had higher eosinophil counts and younger age than those without this presentation (for eosinophil, 17,284 ± 7089 versus 7134 ± 3678/μL, *p* = 0.003; for age 37.5 ± 9.6 versus 47.1 ± 12.5). In the present series, 2 patients expired due to the disease activity, one succumbed to infection complications, and 15 survived with clinical remission.

### 3.2. Myocarditis Characteristics

In Table 2, 10 patients (case nos. 1 to 10), 6 females and 4 males aged 20 to 56 years (40.0 ± 10.1), with negative ANCA in 7, met the diagnostic criteria of overt myocarditis in this study: a presentation at disease onset in 6 and at relapse in 4. Their BVAS were 13 to 37 (25.0 ± 6.8) and FFS were 1 to 3 (1.8 ± 0.6) at the onset of myocarditis. There was lower LVEF (31 to 55%, 42.1 ± 9.2%) with five cases of moderate (case nos. 2, 3, 4, 6 and 10) and five of mild impairment (case nos. 1, 5, 7, 8 and 9). Case nos. 3, 4, 8 and 10 had concurrent pericardial effusion, consistent with the diagnosis of myopericarditis [23], and case nos. 3, 4, 6 and 7 had coexistent endocarditis, an ominous manifestation in EGPA associated with overt heart failure [24]. Notably, patients with additional pericardial or endocardial involvement, indicative of diffuse cardiac involvement [23,25], had lower LVEF than those without such a presentation (for pericarditis, 38.8 ± 7.6% versus 44.3 ± 10.1%; for endocarditis, 38.5 ± 11.2% versus 44.5 ± 7.6%). For myocarditis-related cardiac arrhythmia, case no. 1 had sinus bradycardia complicated with sinus pause, whereas the other 9 patients had sinus tachycardia with additional ventricular extrasystoles in 3 (case nos. 2, 3 and 10) and atrial extrasystoles in 2 (case nos. 7 and 10). All received CSA or plus ART for myocarditis-related cardiac dysfunction.

### 3.3. RTX Treatment

Table 3 shows clinical, laboratory and medication profiles with RTX-related therapeutic indication and regimen in EGPA. Six patients (case nos. 1 to 6) aged 31 to 56 years (44.0 ± 9.0) with 5 seronegativity and FFS 1 to 3 (2.0 ± 0.6), received the RTX treatment without a combined use of CYC at induction, due to refractory disease in 3 patients and relapsing disease in 3. The regimen was 375 mg/m^2^ weekly × 4 intravenous infusions at induction in 6 patients, or plus the same regimen for annual maintenance therapy in 5. Five patients accepted multiple therapeutic courses, 2 to 4 (3 ± 1). All had completely depleted circulating B-cell numbers (0/μL) after induction therapy. They received daily co-trimoxazole prophylaxis against pneumocystitis infection. Seroconversion was found in an ANCA-positive victim, case no. 4, after the RTX treatment. Despite the presence of low immunoglobulin concentrations in 3 patients and infusion reactions in one, there were no infection episodes during the RTX therapeutic period.

There was disease remission with a relapse-free follow-up period of 24 to 67 months (48 ± 15) after completing the RTX induction treatment. Decreased BVAS (21.3 ± 4.6 to 2.2 ± 2.6, *p* = 0.031) were found with a complete remission in 3 (case nos. 1, 2 and 3) and a partial remission in 3 (case nos. 4, 5 and 6). Besides lower CRP levels (26.7 ± 15.1 to 2.7 ± 2.0 mg/L, *p* = 0.031), the eosinophil counts were reduced from 974 ± 185 to 222 ± 130/μL (*p* = 0.031) with a 78 ± 12% inhibition of the baseline values after therapy. Before therapy, the accumulated CYC dosages were beyond 15 g in case nos. 2 and 3, and 10 g in case nos. 4, 5 and 6. After therapy, the CS dosages were reduced to prednisolone 5 mg/day or lower in all, and IS were spared off in 2 patients (case nos. 2 and 5). CSA was prescribed in all patients, in various combinations, for cardiac insufficiency. After therapy, case nos. 2, 3 and 4 only obtained low-dose ACEI/ARB, while case no. 6 received full-dose ACEI.

Case nos. 4 and 6 had a relapsing disease with asthma attacks 18 months and 24 months after completing their last RTX infusion, with a recovery of B-cell numbers to 104/μL and 340/μL, elevated CRP levels to 15.6 mg/L and 11.5 mg/L, higher eosinophil counts to 1,493/μL and 953/μL, and increased BVAS to 9 and 11, respectively (Table 3). In addition, case no. 6 had worsening cardiac insufficiency with increased NYHAFC, raised biomarker concentrations and mildly impaired LVEF, requiring strengthening the use of CSA. Under 100 mg MEP quadri-weekly subcutaneous injection, their asthma presentation was under control. Both had normalized CRP levels, lower eosinophil counts (around 90% inhibition), lower BVAS with a partial remission, and reduced prednisolone dosages to 5 mg/day. Furthermore, case no. 6 had stabilized cardiac function with decreased functional class, lower biomarker concentrations and normalized LVEF.

### 3.4. Therapeutic Efficacy

Table 4 demonstrates the myocarditis-related clinical and rhythm/imaging findings before and after RTX therapy. At onset, all had clinical symptoms, with NYHAFC II in 3 patients and III in 3, cardiac dysrhythmia, elevated concentrations of biomarkers, and lower LVEF with mild impairment in 3, and moderate impairment in 3 (31 to 53%, 38.5 ± 9.3%). LV dilation or global hypokinesia were found in all. Myocardial edema was identified in all, except case no. 4, due to his initial cMRI performed after the completion of two RTX infusion courses. Mid-wall myocardium delayed gadolinium enhancement (DGE) was detected in all. Furthermore, case nos. 3 and 4 had concurrent pericardial effusion (myopericarditis) and case nos. 3, 4 and 6 had coexistent endocardium DGE (endomyocarditis). 

**Figure 2:**
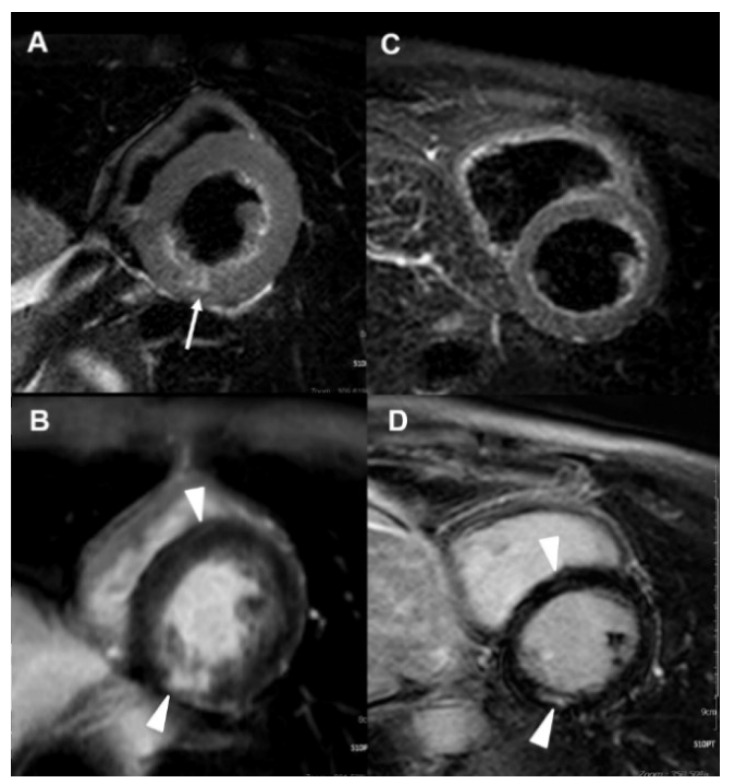
Serial cMRI images in case no. 2 before and after RTX therapy. (**A**,**B**) Pre-RTX treatment cMRI images revealed patchy edema in short-axis T2-weighted images (**A**, white arrows) and post-gadolinium delayed enhancement images (**B**, white arrowheads). (**C**,**D**) After induction and two courses of maintenance therapy, resolved myocardial edema (**C**) and some mid-wall fibrosis at the sepal and inferior walls of LV mid-cavity (**D**, white arrowheads).

**Figure 3:**
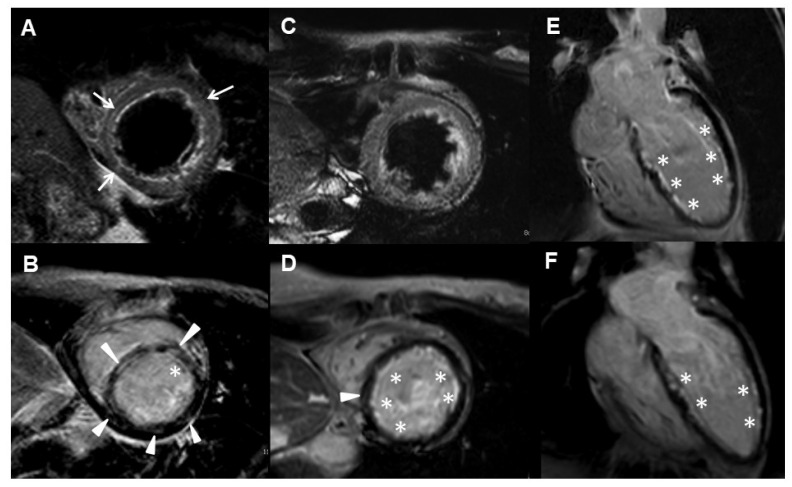
Serial cMRI images in case no. 3 before and after RTX therapy. (**A**,**B**) Pre-RTX treatment cMRI images revealed mid-wall edema in short-axis T2-weighted images (**A**, white arrows) and post-gadolinium delayed enhancement images (**B**, white arrowheads). Regional endocarditis in the anterior wall at LV mid-cavity (**B**, asterisk). (**C**–**E**) After induction therapy, follow-up images showed resolved myocardial edema (**C**), some mid-wall fibrosis (**D**, white arrowheads) and interval worsening of endocarditis (**D**,**E**, asterisks) seen from short-axis and 4-chambered sections. (**F**) The endocardial change revealed improvement after two courses of maintenance therapy (**F**, asterisks).

After RTX therapy, all had improved NYHAFC, normalized biomarkers concentrations (except NT-proBNP in case no. 6), cardiac rhythm, LVEF and LV size/motion, resolved myocardial edema, and reduced myocardium DGE. Although case no. 3 had worsening endocardium DGE after induction, reduced endocardial involvement was found after the maintenance treatment with two RTX therapeutic courses. Nevertheless, case no. 6 had unreduced endocardium DGE after induction therapy. Serial cMRI in case nos. 2 and 3 are shown in Figure 2 and Figure 3, respectively.

RTX was not prescribed in another 4 patients with heart involvement. Shown in Table 2, cardiac manifestation was at disease onset in 3 patients, including myopericarditis (case nos. 8 and 10) and myocarditis alone (case no. 9), and at relapsing disease in one with endomyocarditis (case no. 7). After therapy, case nos. 7, 8 and 9 had clinical remission and stabilized cardiac function under the use of full-dose ACEI/ARB or plus beta blocker (case no. 8). There were decreased functional class, and normalized LVEF, LV size/motion, cardiac dysrhythmia and biomarkers concentrations (case no. 7). Nevertheless, case no. 10 had no clinical improvement with persistent cardiac dysfunction and succumbed to the disease activity with heart failure. 

## 4. Discussion

Although infectious complication is the leading cause of death in the first year after the diagnosis of AAV [26], heart involvement with cardiac insufficiency is another cause of early death and a poor long-term prognostic factor in EGPA [27]. Myocarditis usually presents as non-ischemic cardiomyopathy with heart failure and cardiac arrhythmia [25]. cMRI serves as a non-invasive tool for evaluating the myocardium and endocardium, assessing the extent of heart involvement and helping the evaluation of therapeutic responses [3,23,25]. Combined T2-weighted and post-gadolinium T1-weighted cMRI images can provide the best diagnostic sensitivity and specificity in myocarditis. T2-weighted images are allowed to detect myocardial edema, while T1-weighted DGE can identify myocardial and/or endocardial fibrosis in addition to acute inflammation. The diagnosis of myocarditis and endomyocarditis were based on both T2- and T1-weighted images in this study, with the help of serial follow-up cMRI for evaluating therapeutic responses. Resolved myocardial edema and reduced myocardium DGE were found in all patients after RTX therapy. Furthermore, endocardium DGE was reduced in two patients (case nos. 3 and 4) after induction plus maintenance treatment with two infusion courses, whereas such an abnormality persisted in one (case no. 6) receiving induction therapy alone. Notably, there was a 13% mortality rate in heart involvement under combined CS and CYC induction therapy in an EGPA cohort with the Chinese population [28]. In the present series, our results suggested that administration of RTX might be beneficial in patients with EGPA myocarditis. 

Owing to non-inferiority to CYC, RTX with a 375 mg/m^2^ weekly × 4 regimen was approved as a first-line therapy at induction in severe AAV [1,2]. Since RTX is less toxic than CYC and has lower relapse rates than other IS for maintenance, the 2021 ACR/Vasculitis Foundation Guideline has recommended this biologic agent over CYC for remission induction and over other IS for remission maintenance in active, severe AAV patients [11]. Despite the exclusion of EGPA from AAV trials, the therapeutic effects of RTX at induction in refractory (44%), relapsing (44%) or new-onset disease in EGPA were observed in two retrospective studies with one gram on days 1 and 15 (80%) or 375 mg/m^2^ weekly × 4 regimens [21,29]. Furthermore, a regimen with one gram fortnightly every 6 months has shown efficacy as the maintenance treatment [29]. In addition, a pilot study demonstrated the beneficial outcome in the RTX treatment with multiple therapeutic courses (mean 5 courses) for relapsing disease and remission maintenance [30]. Notably, for active or relapse adult EGPA patients with severe disease, the 2021 ACR/Vasculitis Foundation Guideline has suggested RTX 375 mg/m^2^ weekly × 4 or one gram on days 1 and 15 for remission induction, and 500 mg every 6 months or one gram every 4 months for remission maintenance [11]. In a recent systemic review in RTX-treated EGPA cases, negative-ANCA was found in 35%, while at least two organs were involved and/or neuropathy in 84% of cases [31]. Under the one gram fortnightly regimen given to 61% of patients, complete and partial remission were seen in 53% and 36%, respectively, with infection complications found in 19% of patients. In the present series, therapeutic benefits with 50% complete and 50% partial remission and no infection episodes (all under co-trimoxazole prophylaxis), were observed in overt EGPA myocarditis patients with seronegativity in 83% and under a 375 mg/m^2^ weekly × 4 regimen.

A randomized control trial in EGPA with a regimen of 300 mg MEP subcutaneous injection quadri-weekly for 52 weeks, has demonstrated the efficacy for remission induction in patients with refractory or relapsing disease, thus allowing for a reduction in daily CS dosages [32]. Owing to no specific dose evaluation in that trial, it remains to be determined whether 300 mg is superior to low-dose 100 mg as an EGPA therapy. Subsequent trials with a regimen of 100 mg quadri-weekly were carried out in EGPA patients with a relapsing disease [33,34]. A prospective study with severe disease under 100 mg MEP injection every four weeks for 52 weeks has shown clinical remission with reduced BVAS and decreased prednisolone dosages from 17 to 5 mg/day [33]. Another clinic cohort with patients under the long-term use of prednisolone (average 9 mg/day) received low-dose MEP therapy for 16 weeks, resulting in clinical improvement with completely weaning off CS in all [34]. In this study, a 100 mg quadri-weekly injection regimen was prescribed in two patients (case nos. 4 and 6) for 16 or 24 weeks as the induction treatment for disease relapse with asthma attack, leading to clinical remission with controlled asthma and reduced prednisolone dosage to 5 mg/day. Notably, a European Collaborative Study has recommended a 100 mg dosage as an acceptable first-line therapeutic dose in selected EGPA patients, owing to lacking a comparison with the validated dose of 300 mg [12]. Interestingly, for EGPA in the remission phase, a low-dose regimen was demonstrated to prevent vasculitis relapse with a role of CS/IS sparing agent [35]. 

Although AAV has the presence of ANCA, this autoantibody was identified in 31% of EGPA patients from a French Vasculitis Study Group Cohort [36] and one third of cases in the present series analyzed by ELISA and/or IF methods, implying the existence of two clinical subsets with distinct pathogenic mechanisms based on the ANCA status [1,8]. Seronegative EGPA is less likely to have typical features of other seropositive AAV diseases, while ANCA-negative patients are more susceptible to cardiac involvement [8,37]. Notably, for AAV not including EGPA, neither disease remission nor relapse-free survival after the RTX treatment was shown to be relevant to the seropositivity, suggesting the participation of ANCA-independent working mechanisms in B-cell depleting therapy [38]. Furthermore, elevated peripheral Th17 frequencies and higher serum IgG4 levels were identified in EGPA, especially in correlation with the clinical activity [39,40]. Moreover, RTX therapy was shown to improve disease severity through reducing synovial Th17 numbers in rheumatoid arthritis and induce clinical responses by lowering serum IgG4 concentrations in IgG4-related disease [41,42]. These observations have indicated that the efficacy of RTX treatment in seronegative EGPA patients can be mediated by other action processes, irrelevant to the presence of ANCA, such as through the reduction of Th17 numbers and IgG4 levels.

Heart involvement in EGPA was shown to be associated with higher eosinophil numbers in the peripheral blood than those without the cardiac manifestation [37,43], as also demonstrated by this study in myocarditis patients presenting at the onset of disease. There were lower eosinophil counts in EGPA patients with cardiac involvement after RTX therapy in the present series. In addition to the presence of vasculitis lesions, persistent eosinophilia can cause damage in the myocardium, typically in the form of eosinophilic myocarditis with EGPA as an underlying cause [17]. Furthermore, the endocardium and underlying myocardium are both involved in eosinophilic endomyocarditis, the most characteristic cardiac abnormality in HES [43]. By using cMRI as a survey tool in EGPA, 27% of patients in a case cohort were demonstrated to have endomyocarditis [24]. Since the cMRI examination was only performed in patients with overt heart failure, a lower occurrence (19%) of endomyocarditis was identified in the present series. It is well recognized that heart injury in eosinophilic myocarditis and endomyocarditis is caused by a direct eosinophil-mediated cytotoxicity, eosinophil-degranulation products released from eosinophils, and the recruitment of inflammatory leukocytes by eosinophil-derived cytokines/chemokines [9,17,44]. Notably, anti-IL-5 strategy was proposed to manage eosinophilic myocarditis in HES [17]. The therapeutic efficacy in antagonizing IL-5 was demonstrated in EGPA with cardiac involvement [45,46]. Interestingly, improved cardiac dysfunction after receiving 100 mg MEP quadri-weekly injection was identified in an EGPA patient suffering from eosinophilic myopericarditis with severely impaired LVEF [46]. In this study, an EGPA endomyocarditis victim (case no. 6) with eosinophilia and disease relapse presenting as asthma attack and worsening cardiac insufficiency had controlled asthma and stabilized cardiac function under the same MEP therapeutic regimen.

We demonstrated improved cardiac dysfunction with lower circulating eosinophil numbers after RTX therapy in ANCA-negative and -positive EGPA patients with myocardial or with endocardial involvement in the present series. Indeed, the 2021 ACR/Vasculitis Foundation Guideline has suggested to use RTX for the induction of active or relapse EGPA with severe activity, such as heart involvement [11]. Seroconversion after B-cell-depleting therapy might serve as a working mechanism in treating ANCA-positive EGPA with cardiac presentation [8,31], such as a seropositive endomyocarditis patient (case no. 4) under RTX therapy with multiple infusion courses. Two seropositive EGPA cases with raised serum IL-5 concentrations and increased peripheral eosinophil counts received the RTX treatment, due to their refractory activity to CS and CYC induction, resulting in clinical responses with undetectable IL-5 levels and reduced eosinophil numbers [47]. Furthermore, RTX therapy could improve cutaneous lesions in atopic eczema patients through reducing IL-5 mRNA expression and IL-5-expressing CD4+ T-cell numbers in the skin, lowering Th2 frequencies and eosinophil counts in the blood [48,49]. In addition, in human immunodeficiency virus-associated multicentric Castleman disease with systemic manifestations attributed to disarranged cytokine profiles, there were decreased plasma levels of inflammatory cytokines, including IL-5 after the RTX treatment [50]. Since B cells are involved in the pathogenesis by inducing the release of IL-5 from Th2 cells [6,7,8,9,10], an action mechanism of B-cell-depleting therapy in seronegative EGPA myocarditis might include the suppression of IL-5-mediated eosinophilia.

## 5. Conclusions

In this study, we observed decreased eosinophil counts and improved cardiac dysfunction after B-cell-depleting therapy in seronegative EGPA patients with myocarditis. Rituximab use might have an impact on IL-5-mediated eosinophilia; further mechanistic studies are required to validate this finding.

## Figures and Tables

**Table 1:** Demographic, clinical, laboratory, pathological, medication and outcome data in 21 admitted EGPA patients.

No.	Age ^@^ Sex	Fever ^@^	Skin	Sinus	Joint	Mus	Lung	Heart	GI	Renal	PNS	CNS	FFS ^@^	BVAS ^@^	Pathology Findings	CRP mg/L ^@^	Eosin /μL ^@^	ANCA Status ^@^	ACR Item ^@^	Medication Profile	Final Outcome
																				CS/CYC/AZA/MTX/RTX	
1	45 M	Yes	Yes	Yes	Nil	Nil	Yes	Yes	Yes	Nil	Yes	Nil	1	31	Eosinophilia Vasculitis	51.1	6090	Negative	6	Yes/Yes/Yes/Nil/Yes	Survival, remission
2	30 M	Nil	Yes	Yes	Nil	Yes	Yes	Yes	Yes	Yes	Yes	Nil	2	36	Eosinophilia Vasculitis	92.6	11,567	Negative	6	Yes/Yes/Yes/Nil/Yes	Survival, remission
3	45 F	Nil	Yes	Nil	Yes	Yes	Yes	Yes	Nil	Yes	Yes	Nil	2	29	Eosinophilia Vasculitis	46.5	16,947	Negative	5	Yes/Yes/Yes/Nil/Yes	Survival, remission
4	36 M	Yes	Yes	Yes	Yes	Yes	Yes	Yes	Yes	Nil	Yes	Nil	2	37	Eosinophilia Vasculitis	171.9	17,424	Positive, anti-MPO	6	Yes/Yes/Yes/Nil/Yes	Survival, remission
5	47 F	Yes	Nil	Yes	Nil	Nil	Yes	Yes	Nil	Nil	Yes	Nil	1	30	Eosinophilia	183.3	26,781	Negative	6	Yes/Yes/Yes/Nil/Yes	Survival, remission
6	55 F	Yes	Yes	Nil	Yes	Nil	Yes	Yes	Nil	Nil	Yes	Nil	1	28	Eosinophilia Vasculitis	165.1	5806	Negative	5	Yes/Yes/Yes/Nil/Yes	Survival, remission
7	39 F	Nil	Yes	Nil	Nil	Nil	Yes	Yes	Nil	Nil	Yes	Nil	1	20	Eosinophilia	39.8	10,140	Positive, anti-MPO	5	Yes/Yes/Yes/Nil/Nil	Survival, remission
8	40 M	Yes	Yes	Yes	Nil	Nil	Yes	Yes	Nil	Nil	Yes	Nil	1	24	Eosinophilia Vasculitis	47.5	23,392	Negative	6	Yes/Yes/Yes/Nil/Nil	Survival, remission
9	37 F	Nil	Yes	Yes	Yes	Nil	Yes	Yes	Nil	Nil	Nil	Nil	1	19	Eosinophilia	42.5	11,139	Positive, anti-MPO	5	Yes/Yes/Yes/Nil/Nil	Survival, remission
10	20 F	Yes	Yes	Nil	Nil	Yes	Yes	Yes	Nil	Nil	Yes	Yes	2	26	Eosinophilia Vasculitis	98.8	8019	Negative	5	Yes/Yes/Yes/Nil/Nil	Death due to activity
11	47 M	Yes	Nil	Nil	Nil	Nil	Yes	Nil	Nil	Yes	Yes	Nil	1	30	Eosinophilia	54.8	9750	Positive, anti-MPO	5	Yes/Yes/Yes/Nil/Nil	Survival, remission
12	29 F	Yes	Yes	Nil	Nil	Nil	Yes	Nil	Nil	Nil	Yes	Nil	1	21	Eosinophilia Vasculitis	80.7	12,816	Negative	5	Yes/Nil/Yes/Yes/Nil	Survival, remission
13	66 M	Nil	Nil	Yes	Yes	Nil	Yes	Nil	Nil	Nil	Yes	Nil	1	20	Eosinophilia	76.3	2541	Negative	6	Yes/Nil/Yes/Nil/Nil	Survival, remission
14	70 M	Nil	Yes	Yes	Nil	Nil	Nil	Nil	Nil	Nil	Yes	Nil	1	21	Eosinophilia	44.8	3744	Negative	5	Yes/Nil/Yes/Nil/Nil	Survival, remission
15	32 M	Yes	Yes	Nil	Yes	Yes	Yes	Nil	Nil	Yes	Yes	Nil	2	39	Eosinophilia Vasculitis	80.1	2314	Positive, anti-MPO	5	Yes/Yes/Yes/Nil/Nil	Death due to activity
16	56 F	Nil	Nil	Yes	Yes	Nil	Yes	Nil	Yes	Nil	Yes	Nil	1	25	Eosinophilia	70.0	13,029	Negative	5	Yes/Yes/Yes/Nil/Nil	Survival, remission
17	49 F	Yes	Yes	Yes	Nil	Nil	Yes	Nil	Yes	Nil	Nil	Nil	1	21	Eosinophilia	32.1	4429	Negative	5	Yes/Yes/Yes/Nil/Nil	Survival, remission
18	54 M	Yes	Nil	Yes	Nil	Nil	Yes	Nil	Nil	Yes	Yes	Yes	1	32	Eosinophilia	61.9	8775	Positive, anti-MPO	6	Yes/Yes/Yes/Nil/Nil	Death due to infection
19	38 M	Nil	Nil	Yes	Yes	Nil	Yes	Nil	Yes	Nil	Yes	Nil	1	27	Eosinophilia	62.1	7696	Negative	6	Yes/Yes/Yes/Yes/Nil	Survival, remission
20	41 M	Nil	Yes	Nil	Yes	Yes	Yes	Nil	Nil	Nil	Yes	Nil	1	23	Eosinophilia	29.4	3952	Positive, anti-MPO	5	Yes/Yes/Yes/Nil/Nil	Survival, remission
21	55 M	Nil	Yes	Nil	Yes	Nil	Yes	Nil	Nil	Nil	Yes	Nil	1	18	Eosinophilia	32.1	4356	Negative	5	Yes/Yes/Yes/Nil/Nil	Survival, remission
Stat ^#^	F 43% 44 ± 12	52%	71%	57%	48%	29%	95%	48%	29%	24%	95%	10%	1.3 ± 0.4	27 ± 6	Vasculitis 43%	74.9 ± 45.5	10,034 ± 6641	Negative 67%	5.4 ± 0.5	100%/86%/100%/10%/29%	Survival 86%

^@^ Age, FFS, BVAS, ACR item at the EGPA diagnosis, and Fever, CRP, Eosin, ANCA at the disease onset, ^#^ Numerical data as the mean and standard deviation. ACR: American College of Rheumatology, ANCA: anti-neutrophil cytoplasmic antibody, AZ: azathioprine, BVAS: Birmingham Vasculitis Activity Score, CNS: central nervous system, CS: corticosteroids, CYC: cyclophosphamide, EGPA: eosinophilic granulomatosis with polyangiitis, Eosin: eosinophil, F: female, FFS: five-factor score, GI: gastrointestinal, M: male, MPO: myeloperoxidase, MTX: methotrexate, Mus: muscle, No.: number, PNS: peripheral nervous system, RTX: rituximab, Stat: statistics.

**Table 2:** Clinical, medication and outcome profiles before and after therapy in 10 EGPA patients with myocarditis.

No.	Age ^@^ Sex	FFS ^@^/ BVAS ^@^	ANCA Status before/after	Myocarditis Type/Onset	NYHAFC, Rhythm before/after	Biomarker before/after	Impaired LVEF before/after	Cardiac Therapeutics before/after	Disease Outcome
1	47 M	2/23	Negative/ Negative	Myocarditis/ Disease relapse	II/I SB, SP/NSR	Elevated/ Normalized	Mildly/ Normalized	Combined CSA/ Nil	Complete remission
2	31 M	3/29	Negative/ Negative	Myocarditis/ Disease relapse	III/I ST, PVC/NSR	Elevated/ Normalized	Moderately/ Normalized	Combined CSA, ART/ low-dose ARB	Complete remission
3	46 F	2/20	Negative/ Negative	Endomyocarditis Myopericarditis/ Disease onset	III/I ST, PVC/NSR	Elevated/ Normalized	Moderately/ Normalized	Combined CSA, ART/ low-dose ACEI	Complete remission
4	36 M	2/15	Positive/ Negative	Endomyocarditis Myopericarditis/ Disease onset	III/I ST/NSR	Elevated/ Normalized	Moderately/ Normalized	Combined CSA/ low-dose ACEI	Partial remission
5	48 F	1/21	Negative/ Negative	Myocarditis/ Disease onset	II/I ST/NSR	Elevated/ Normalized	Mildly/ Normalized	Combined CSA/ Nil	Partial remission
6	56 F	2/20	Negative/ Negative	Endomyocarditis/ Disease relapse	III/II ST/NSR	Elevated/ Normalized ^#^	Moderately/ Normalized	Combined CSA /ACEI	Partial remission
7	39 F	2/13	Positive/ Negative	Endomyocarditis/ Disease onset	II/I ST, PAC, PAT/NSR	Elevated/ Normalized	Mildly/ Normalized	Combined CSA, ART /ACEI	Complete remission
8	40 M	1/24	Negative/ Negative	Myopericarditis/ Disease onset	II/I ST/NSR	Elevated/ Normalized ^#^	Mildly/ Normalized	Combined CSA/ Combined CSA	Partial remission
9	37 F	1/19	Positive/ Negative	Myocarditis/ Disease onset	II/I ST/NSR	Elevated/ Normalized ^#^	Mildly/ Normalized	Combined CSA/ ARB	Partial remission
10	20 F	2/26	Negative/ Negative	Myopericarditis/ Disease onset	III/IV ST, PAC, PVC/ST, PVC	Elevated/ Elevated	Moderately/ Severely	Combined CSA, ART/ Combined CSA, ART	Death due to heart failure

^@^ At the myocarditis onset, ^#^ Normalized other biomarkers with decreased NT-proBNP levels. ACEI: angiotensin converting enzyme inhibitor, ANCA: anti-neutrophil cytoplasmic antibody, ARB: angiotensin-receptor blocker, ART: antiarrhythmics, BVAS: Birmingham Vasculitis Activity Score, CSA: cardiac supportive agents, EGPA: Eosinophilic granulomatosis with polyangiitis, F: female, M: male, FFS: five-factor score, No.: number, NYHAFC: New York Heart Association Functional Classification, LVEF: left ventricle ejection fraction, NSR: normal sinus rhythm, PAC: paroxysmal atrial tachycardia, PAT: paroxysmal atrial tachycardia, PVC: premature ventricular contraction, SB: sinus bradycardia, SP: sinus pause, ST: sinus tachycardia.

**Table 3:** Clinical, laboratory, medication, response data and RTX indication/regimen in six EGPA myocarditis patients.

No.	Age Sex /FFS	ANCA before/ after RTX	Biologics Indication	Biologics Regimen (Course)	^@^ B Cell (/μL) Diff	^@^ BVAS Diff	^@^ Eosinophil (/μL) Diff (Inh %)	^@^ CRP (mg/L) Diff	Side Effects	^#^ Follow Up Time	Biologics Therapeutic Response	CS and IS before/after Biologics
1	47 M 2	Negative/ negative	Induction for disease relapse /maintenance	375 mg/m^2^ weekly × 4 RTX (4)	35 to 0	23 to 0	1170 to 149 (87.3%)	24.0 to 1.8	Low IgM	67 m	Complete remission	AZ, CS, CYC /AZ, ^+^ low-dose CS
2	31 M 3	Negative/ negative	Induction for disease relapse /maintenance	375 mg/m^2^ weekly × 4 RTX (3)	103 to 0	29 to 0	826 to 95 (88.5%)	8.0 to 2.2	Low IgG/M	56 m	Complete remission	AZ, CS, CYC /^+^ low-dose CS
3	46 F 2	Negative/ negative	Induction for refractory /maintenance	375 mg/m^2^ weekly × 4 RTX (3)	71 to 0	20 to 0	882 to 159 (82.0%)	23.0 to 1.1	Nil	53 m	Complete remission	CS, CYC /AZ, ^+^ low-dose CS
4	36 M 2	Positive/ negative Negative/ ND	Induction for refractory /maintenance Induction for disease relapse	375 mg/m^2^ weekly × 4 RTX (3), 100 mg quadri-weekly × 4 MEP	ND to 0 104 to ND	15 to 4 9 to 3	1206 to 322 (73.3%) 1,493 to 164 (89.0%)	17.9 to 3.2 15.6 to 1.9	Nil Nil	42 m 4 m	Partial remission after RTX, relapse at 43th m Partial remission after MEP	CS, CYC /AZ, ^+^ low-dose CS CS /^+^ low-dose CS
5	48 F 1	Negative/ negative	Induction for refractory /maintenance	375 mg/m^2^ weekly × 4 RTX (2)	159 to 0	21 to 3	755 to 172 (77.2%)	51.6 to 1.5	Low IgM	44 m	Partial remission	CS, CYC /^+^ low-dose CS
6	56 F 2	Negative/ Negative Negative/ negative	Induction for disease relapse Induction for disease relapse	375 mg/m^2^ weekly × 4 RTX (1), 100 mg quadri-weekly × 6 MEP	316 to 0 340 to 329	20 to 6 11 to 4	1006 to 437 (56.6%) 953 to 92 (90.3%)	35.4 to 6.4 11.5 to 1.6	Infusion reaction 1st dose Nil	24 m 6 m	Partial remission after RTX, relapse at 25th m Partial remission after MEP	AZ, CS, CYC /AZ, ^+^ low-dose CS CS /^+^ low-dose CS

^@^ Calculation before and after biologic use, ^#^ Follow-up time after completing RTX induction or starting MEP therapy, ^+^ low-dose CS, 5 mg/day prednisolone or lower dosages. ANCA: anti-neutrophil cytoplasmic antibody, AZ: azathioprine, CS: corticosteroid, CYC: cyclophosphamide, Diff: difference, EGPA: eosinophilic granulomatosis with polyangiitis, F: female, FFS: five-factor score, Inh: inhibition, IS: immunosuppressants, m: month, M: male, MEP: mepolizumab, ND: not done, No.: number, RTX; rituximab, yr: year.

**Table 4:** Cardiac symptom, rhythm, biomarker and image data in six EGPA myocarditis patients receiving RTX therapy.

No.	Involved Cardiac Area	Symptoms, NYHAFC before/after	Rhythm before/ after	Biomarkers before/after	Image Findings of cMRI and TTE before (^@^ During) RTX Therapy	Image Findings of cMRI and ECG after RTX Therapy
1	Myocardium	Dyspnea /Nil II/I	SB with SP /NSR	Elevated /Normalized	Dilated LV Mildly impaired LVEF Myocardial edema Multifocal mid-wall DGE at basal LV, LV mid-cavity IVS	Normalized LV size Normalized LVEF Resolved myocardial edema Reduced mid-wall DGE
2	Myocardium	Dyspnea, orthopnea, palpitation /Nil III/I	ST with PVC /NSR	Elevated /Normalized	Dilated LV with global hypokinesia Moderately impaired LVEF Myocardial edema Patchy mid-wall DGE at LV mid-cavity IVS and inferolateral wall, apical LV anterolateral wall	Normalized LV size and motion Normalized LVEF Resolved myocardial edema Reduced mid-wall DGE
3	Endocardium, myocardium, pericardium	Dyspnea, orthopnea, chest pain /Nil III/I	ST with PVC /NSR	Elevated /Normalized	LV global hypokinesia Moderately impaired LVEF Pericardial effusion Myocardial edema Curvilinear mid-wall DGE at global LV Diffuse endocardial DGE at global LV	Normalized LV motion Normalized LVEF Resolved pericardial effusion Resolved myocardial edema Reduced mid-wall DGE Reduced endocardial DGE
4	Endocardium, myocardium, pericardium	Dyspnea, orthopnea, chest pain /Nil III/I	ST /NSR	Elevated /Normalized	LV global hypokinesia Moderately impaired LVEF Pericardial effusion ^@^ Resolved myocardial edema ^@^ Spotty mid-wall DGE at basal LV inferolateral wall ^@^ Endocardial DGE at basal LV, LV mid-cavity	Normalized LV motion Normalized LVEF Resolved pericardial effusion Resolved myocardial edema Reduced mid-wall DGE Reduced endocardial DGE
5	Myocardium	Dyspnea /Nil II/I	ST /NSR	Elevated /Normalized	Dilated LV Mildly impaired LVEF Myocardial edema Curvilinear mid-wall DGE at basal LV anteroseptal wall, spotty mid-wall DGE at LV mid-cavity antero-lateral wall	Normalized LV size Normalized LVEF Resolved myocardial edema Reduced mid-wall DGE
6	Endocardium, myocardium	Dyspnea, orthopnea, palpitation /Dyspnea III/II	ST /NSR	Elevated /^#^ Normalized	LV global hypokinesia Moderately impaired LVEF Myocardial edema Curvilinear mid-wall DGE at basal LV, LV mid-cavity Endocardial DGE at LV mid-cavity	Normalized LV motion Normalized LVEF Resolved myocardial edema Reduced mid-wall DGE Unreduced endocardial DGE

^@^ cMRI done before initiating RTX therapy in all, except case no. 4, before the 3rd infusion course; ^#^ Normalized other biomarkers with decreased NT-proBNP levels. cMRI: cardiac magnetic resonance imaging, DGE: delayed gadolinium enhancement, EGPA: eosinophilic granulomatosis with polyangiitis, IVS: interventricular septum, LVEF: left ventricle ejection fraction, No.: number, NSR: normal sinus rhythm, NT-proBNP: N-terminal pro-brain natriuretic peptide, PAC: premature atrial contraction, PAT: paroxysmal atrial tachycardia, PVC: premature ventricular contraction, RTX: rituximab, SB: sinus bradycardia, SP: sinus pause, ST: sinus tachycardia, TTE: transthoracic echocardiography.

## Data Availability

The data of this study can be provided to researchers from the corresponding author upon request.
